# SDQ in the Hands of Fathers and Preschool Teachers—Psychometric Properties in a Non-clinical Sample of 3–5-Year-Olds

**DOI:** 10.1007/s10578-018-0826-4

**Published:** 2018-06-29

**Authors:** Anton Dahlberg, Ata Ghaderi, Anna Sarkadi, Raziye Salari

**Affiliations:** 1 Child Health and Parenting (CHAP), Department of Public Health and Caring Sciences, Uppsala University, Box 564, Uppsala, 751 22 Sweden; 2 Department of Clinical Neuroscience, Division of Psychology, Karolinska Institutet, Stockholm, Sweden

**Keywords:** Strengths and Difficulties Questionnaire (SDQ), Fathers, Confirmatory factor analysis (CFA), Preschool children, Construct validity

## Abstract

The Strengths and Difficulties Questionnaire (SDQ) is a well-established instrument for measuring social and behavioural problems among children, with good psychometric properties for older children, but less validity reports on pre-schoolers. In addition, there is a knowledge gap concerning fathers as informants. The present work is one of the few validity studies to include preschool teachers and the first on preschool children where fathers are included as separate informants. In this study, SDQs were collected from a large community sample (*n* = 17,752) of children aged 3–5, rated by mothers, fathers, and preschool teachers and analysed using confirmatory factor analysis. Our results revealed acceptable fit for all informant groups and measurement invariance across child gender, child age, and parental education level. Our findings suggest good construct validity of the SDQ for a non-clinical preschool population and imply that it may be used for assessing child behaviour problems from different informant perspectives.

## Introduction

In research involving children, there is general agreement on the importance of early discovery of and early interventions towards mental health problems. For this to be possible, we need instruments with proper psychometric qualities. One of the most commonly used instruments for assessing behaviour and mental health problems among younger children is the Strengths and Difficulties Questionnaire (SDQ), which is being used both clinically and in research on children aged 2–17 (http://www.sdqinfo.com). Consisting of 25 items, the SDQ is a relatively short questionnaire while still being comparable to the similar but lengthier Child Behavior Checklist (CBCL), displaying moderate to high correlations on total and equivalent subgroup scores [1, 2]. The SDQ, in general, displays good construct and concurrent validity, as well as some evidence on predictive validity [3–5]. Construct validity has mainly been assessed using confirmatory factor analysis (CFA). Although displaying promising psychometric properties, few studies have investigated the SDQs validity and reliability for use among preschool-aged children specifically (see Croft et al. [6] for one example); on the contrary, it has mostly been used with teenagers or a broad range of ages spanning through preschool and the early school years. A study by Croft et al. [6] concluded satisfactory construct validity for preschool children when rated by parents.

Factor analytic studies of the SDQ have supported the original five-factor structure in many [7–10] but not all cases [5, 11, 12]. In a large sample study, Goodman et al. [13] concluded that the five-factor model should be used in clinical samples, while a model with two broader externalising and internalising subscales should be used in epidemiological studies or low-risk samples. Stone et al. [3] found support for the original factor solution, when analysing data from several previous studies. To the best of our knowledge, the major part of the CFA studies published concern school children. Thus, we identified a need for more studies on the construct validity of the SDQ for preschool children. Ezpeleta et al. [14] have provided some evidence suggesting that the original five-factor model is feasible for preschool children, although not entirely convincing mainly due to low values on indices of comparative fit. Investigating factor structure of the SDQ for pre-schoolers in a Nordic context, support for a two-factor model consisting of hyperactivity and conduct problems was found for children aged 1–3, while a model including the original subscales, except for the prosocial subscale, was proposed for 4–5-year-olds [15].

The multi-informant approach of the SDQ provides opportunity to assess the validity of the questionnaire when filled in by different informant groups. Data from teachers’ ratings generally show better model fit than parents, especially on subscale level [3]. Although there is evidence for good validity of teachers’ SDQ ratings of children in primary school [16], no CFA studies have included preschool teachers.

Some studies of the SDQ have indicated that fathers tend to report more externalising behaviour problems than mothers [17, 18]. Interestingly, fathers are rarely treated as a unique informant group in validity studies of the SDQ. In the published CFA studies that we found, separate analyses of mothers and fathers are conspicuous by their absence, with very few exceptions (e.g. [19]). Looking at how parent data are constituted, mothers are greatly overrepresented, with fathers mostly present as either co-respondents or marginally represented together with the mothers (e.g. [14, 20]). It is also common to report parents as one group, not specifying the proportions of mothers and fathers (e.g. [16, 21]). Although it can be argued that the factor structure of the SDQ rated by fathers and mothers should be similar—a statement somewhat supported by Björnsdotter et al. [19]—this is something that needs to be empirically investigated further in order to answer questions regarding the validity of SDQ across different informants and age groups.

For preschool children, measurement invariance has been established between mothers and fathers [22]. Still, to the best of our knowledge, the only studies assessing invariance across different age groups, child gender, or parental (maternal) education level within each informant group are on school-aged children [23].

The aim of the present study is to test the original five-factor structure of the SDQ for preschool children and to assess whether the suggested model has an acceptable fit for fathers as well as mothers and preschool teachers. This study also seeks to assess measurement invariance across child gender, child age, and parental education for all three informant groups.

## Methods

### Data Collection

For this study, data were extracted from the *Children and Parents in Focus* project [24]: an ongoing population-based intervention trial in Uppsala, Sweden, aiming at investigating the mental health of preschool children and their parents, and evaluating the effects of a parenting programme. Parents and preschool teachers filled in a set of questionnaires in connection with the children’s annual check-up at Child Health Centres, including the SDQ. Since more than 90% of all children aged 3–5 in Sweden attend preschool [25] and 95% visit the child health centres regularly [26], a major part of the population was targeted.

For detailed background, measures, study design, and field procedures, see Salari et al. [24]. The extracted data were collected between August 2013 and August 2016. Since data were collected during a 3-year period for all children aged 3–5 in the same geographical area, some children were represented in the data set at two or three different ages. The study was approved by the Regional Ethical Review Board in Uppsala (Dnr 2012/437). Informed consent was obtained from all participants included in the study.

### Sample

**Fig. 1:**
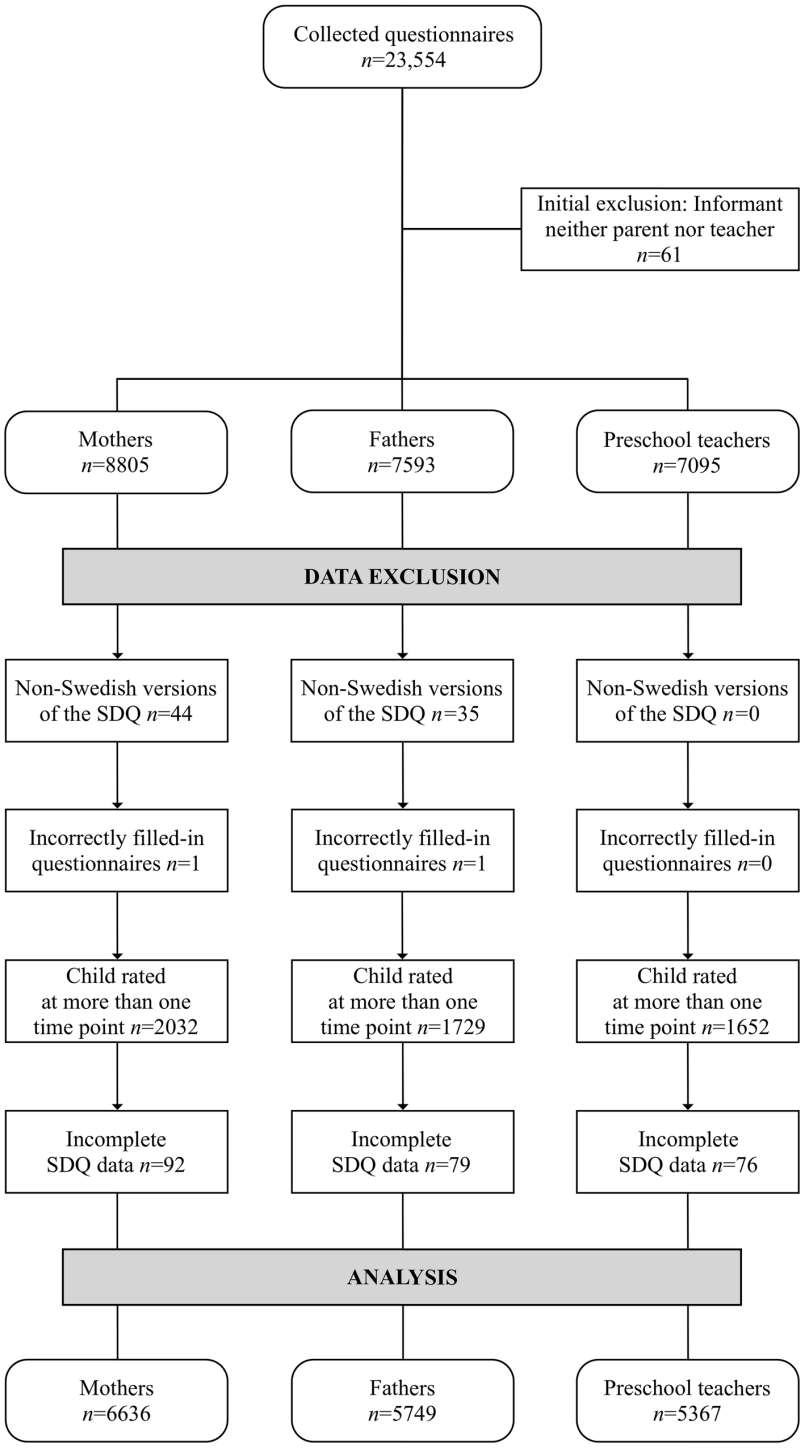
Flowchart of exclusion

A total of 23,554 questionnaires were collected from parents and teachers of pre-schoolers aged 3–5. For the study at hand, we excluded data where informants were not the mother, father or preschool teacher of the focal child; when the questionnaires were completed in languages other than Swedish; and when more than one child was rated on the same questionnaire. For children who were represented at two or three time points during the study, one questionnaire was selected at random. Finally, to assess subscale scores on the SDQ, at least three items per subscale need to be filled in (http://www.sdqinfo.com). Therefore, questionnaires with insufficient amount of data based on these restrictions were also excluded from statistical analyses. After data exclusion, questionnaires from 6636 mothers, 5749 fathers, and 5367 preschool teachers, representing 7113 children, remained for statistical analyses. The order of exclusion and number of excluded cases are displayed in Fig. 1. All three informant groups had an equal proportion of children from each age group present: the percentages of 3-, 4-, and 5-year-olds were 34, 32, and 32%, respectively. Girls and boys were equally represented across all three informant groups. Information on child gender was missing from three questionnaires from fathers, eight questionnaires from mothers, and from one questionnaire from preschool teachers. The proportions of the 7113 children rated by different combinations of informants are displayed in Table 1.

**Table 1:** Proportions of children rated by different combinations of informants

	One parent	Preschool teacher	Both parents	One parent and teacher	All three informants	Total
*n*	741	80	1005	940	4347	7113
%	10.4	1.1	14.1	13.2	61.1	100

The age of the parents ranged between 19 and 58 for mothers (*M* = 35.8, SD = 4.9) and between 19 and 68 for fathers (*M* = 38.3, SD = 5.9). To assess education level, parents were divided into two categories: less than 3 years of college or university education, and more than 3 years of college or university education. The number of parents with college or university education of at least 3 years was compared with municipality data retrieved from Statistics Sweden (scb.se). Comparisons revealed a skewness of the data towards higher education level: 52% of the fathers and 63% of the mothers in our sample compared with 49% within the municipality. Non-native parents comprised 14% of the fathers and 14% of the mothers, which was representative of the municipality population.

### Instruments

The SDQ consists of 25 items, each rated as being *not true* (0), *somewhat true* (1), or *certainly true* (2). Items are divided into five subscales covering conduct problems, hyperactivity, emotional symptoms, peer problems, and prosocial behaviour [27]. Summing up the scores on the first four subscales generates the SDQ total difficulties score, ranging from 0 to 40.

The Swedish version of the SDQ was used to collect data from children 3–5 years old. Some items were slightly changed in wording after discussions with health, research, and pedagogy professionals involved in the Children and Parents in Focus study (see Table 2 for the SDQ items and modifications). The reason for this was that the preschool professionals considered the original wording of some SDQ items to be disharmonious with their philosophical and pedagogical beliefs and thus refused to respond if they were not altered. Thus, we agreed on a clearer focus on behaviours rather than specific traits of individual children. The altered wording of the three items in question were considered to be in line with both pedagogical praxis in Sweden, and with the original intention of creating a questionnaire focusing on displayed behaviour [27]. Demographic information about the child (e.g. birthdate and gender) and the parent (gender, education level) were collected together with the SDQ.

**Table 2:** The original five-factor structure of the SDQ

Subscale	Items
Conduct problems	Often has temper tantrums or hot tempers
	Generally obedient, usually does what adults request^a^
	Often fights with other children or bullies them
	Often argumentative with adults
	Can be spiteful to others^b^
Hyperactivity	Restless, overactive, cannot stay still for long
	Constantly fidgeting or squirming
	Easily distracted, concentration wanders
	Can stop and think things out before acting
	Sees tasks through to the end, good attention span
Emotional symptoms	Often complains of headaches, stomach-aches or sickness
	Many worries, often seems worried
	Often unhappy, down-hearted or tearful
	Nervous or clingy in new situations, easily loses confidence
	Many fears, easily scared
Peer problems	Rather solitary, tends to play alone
	Has at least one good friend
	Generally liked by other children
	Picked on or bullied by other children
	Gets on better with adults than with other children
Prosocial behaviour	Considerate of other people’s feelings
	Shares readily with other children (treats, toys, pencils, etc.)
	Helpful if someone is hurt, upset or feeling ill
	Kind to younger children^c^
	Often volunteers to help others (parents, teachers, other children)

Modified items are presented in the footnotes

^a^Usually does what adults request

^b^Can behave spitefully towards others

^c^Considerate of younger children

### Procedure

All parents of children aged 3–5 were invited to fill in the questionnaires as part of their annual check-up at child health centres. Along with the invitation letter to the annual check-up, three sets of questionnaires were sent home to each child. Parents/guardians were asked to fill in one questionnaire each and bring the completed forms to the visit. In addition, parents were instructed to take the third questionnaire to the child’s preschool and ask the preschool teacher to complete the form, put it in the prepaid envelope provided and send it directly to the child health centre. The preschool teachers in Sweden have a three-and-a-half-year academic education and have professional knowledge in child development and pedagogical interventions aimed at children aged 1–6.

### Statistical Analysis

All statistical analyses were conducted using R, version 3.4.1 [28]. The items of the SDQ were rated on a 3-point Likert scale and thus treated as ordinal data. Therefore, internal consistency was assessed based on polychoric ordinal alpha calculations as proposed by Gadermann et al. [29], using the psych package [30]. The fit of the original theoretical five factor model of the SDQ was assessed through CFA, using the lavaan package [31]. Due to the ordinal nature of the data, analyses were based on polychoric correlation matrices, using Diagonally Weighted Least Squares (DWLS) for estimation of model parameters [32]. Chi square (with alpha set to *p* < .05), Root Mean Square Error of Approximation (RMSEA), Comparative Fit Index (CFI), and Tucker-Lewis Index (TLI) were used to test model fit. The criteria for acceptable model fit were set to RMSEA less than 0.06, in combination with CFI or TLI above 0.90 [33]. Although the Chi square statistics were calculated, they are of little importance as a measure of model fit in our analyses, since the large sample sizes make it very likely to find significant differences between models [34, 35]. Because the majority of the children were rated by more than one informant, three separate analyses were conducted for fathers, mothers, and preschool teachers.

The questionnaire used was designed for children aged 2–4, and the children in our study were aged 3–5. In order to determine whether the questionnaire was still psychometrically valid, measurement invariance (MI) analysis was applied to assess potential differences across age groups. This was also applied for child gender and parental education level. These analyses were carried out within the CFA framework, imposing equality constraints to the factor loadings and thresholds in a hierarchal manner. First, a free model with no equality constraints was analysed to assess fitness for each subgroup. Secondly, a model with constrained factor loadings was specified and compared to the free model. If the models were not significantly different in fit, a third model with constrained loadings and intercepts was specified and compared to the second model. Comparing the restricted and unrestricted models, changes in *χ*^2^ are often used as a measure. However, given the large sample sizes, even very small changes are likely to be significant, making this measure unsuitable for our analyses [35]. Instead, change in CFI was used as a subgroup invariance measure, as proposed by Cheung and Rensvold [36] and Chen [37], and applied by He et al. [5]. Changes in CFI less than 0.01 were considered not significant.

## Results

### Internal Consistency

The calculated alpha values indicated good internal consistency for fathers (conduct problems: 0.78, hyperactivity: 0.84, emotional symptoms: 0.72, peer problems: 0.72, prosocial behaviour: 0.82, and total difficulties: 0.87), mothers (conduct problems: 0.79, hyperactivity: 0.87, emotional symptoms: 0.73, peer problems: 0.77, prosocial behaviour: 0.84, and total difficulties: 0.88), and preschool teachers (conduct problems: 0.88, hyperactivity: 0.92, emotional symptoms: 0.81, peer problems: 0.84, prosocial behaviour: 0.92, and total difficulties: 0.92).

### Factor Structure

Model fit indices from the CFAs of mothers, fathers, and preschool teachers are presented in Table 3. All fit indices for the five-factor model were satisfactory, suggesting acceptable fit for all groups (CFI and TLI > 0.90 and RMSEA < 0.06). This suggested that the original factor structure was feasible for use on younger children, rated by fathers, mothers or preschool teachers.

**Table 3:** Model fit for confirmatory factor analyses for different informants

	*n*	Model Fit Indices			
		*χ*^2^ (df)	CFI	TLI	RMSEA (90% CI)
Fathers	5749	3510.038 (265)	0.914	0.902	0.049 (0.048–0.050)
Mothers	6636	3487.356 (265)	0.926	0.916	0.046 (0.045–0.048)
Preschool teachers	5367	3146.890 (265)	0.953	0.947	0.050 (0.048–0.051)

*CFI* Comparative Fit Index, *TLI* Tucker-Lewis Index, *RMSEA* root mean square error of approximation, *CI* confidence interval

CFI or TLI > 0.90 and RMSEA < 0.06 indicate acceptable fit

### Measurement Invariance

Assuming adequate fit for all three informant groups, subgroup CFAs were conducted to assess model fit based on child gender, child age, and parental education level. Results (Tables 4, 5) indicated acceptable fit for all subgroups. Following these analyses, multiple-group CFAs were conducted across all sub-populations to assess MI. When imposing equality constraints to factor loadings within child gender subgroups, no significant change in model fit was detected for fathers, mothers, or preschool teachers (Table 4). This implied metric invariance, meaning that the items on the SDQ measure the latent factors comparably for girls and boys. Next, when imposing equality constraints to the thresholds, no significant change in model fit was detected for fathers, mothers, or preschool teachers (Table 4), implying scalar invariance, or that the meaning of the subscales and the levels of the underlying items are equal across the child gender subgroups.

**Table 4:** Model fit and nested model comparisons for multiple-group CFAs: child gender and child age

Multiple group CFA	Model Fit Indices			Nested model comparisons	
	CFI	TLI	RMSEA (90% CI)	Comparison	CFI change
Fathers					
Child gender^†^					
Girls (*n* = 2791)	0.905	0.893	0.048 (0.046–0.050)		
Boys (*n* = 2955)	0.921	0.910	0.048 (0.046–0.050)		
Model 1^a^	0.914	0.902	0.048 (0.046–0.049)		
Model 2^b^	0.921	0.914	0.045 (0.044–0.046)	Model 2 vs 1	0.007
Model 3^c^	0.916	0.912	0.046 (0.044–0.047)	Model 3 vs 2	0.005
Child age					
3-year-olds (*n* = 1955)	0.910	0.899	0.047 (0.045–0.050)		
4-year-olds (*n* = 1828)	0.909	0.897	0.048 (0.045–0.051)		
5-year-olds (*n* = 1966)	0.926	0.917	0.045 (0.042–0.048)		
Model 1^a^	0.916	0.905	0.047 (0.045–0.048)		
Model 2^b^	0.925	0.919	0.043 (0.042–0.045)	Model 2 vs 1	0.009
Model 3^c^	0.918	0.916	0.044 (0.043–0.045)	Model 3 vs 2	0.007
Mothers					
Child gender^‡^					
Girls (*n* = 3224)	0.917	0.906	0.044 (0.042–0.046)		
Boys (*n* = 2955)	0.921	0.910	0.048 (0.046–0.050)		
Model 1^a^	0.927	0.917	0.045 (0.044–0.047)		
Model 2^b^	0.933	0.927	0.043 (0.041–0.044)	Model 2 vs 1	0.006
Model 3^c^	0.928	0.925	0.043 (0.042–0.045)	Model 3 vs 2	0.005
Child age					
3-year-olds (*n* = 2257)	0.929	0.919	0.044 (0.041–0.046)		
4-year-olds (*n* = 2100)	0.916	0.905	0.047 (0.045–0.050)		
5-year-olds (*n* = 2279)	0.939	0.930	0.042 (0.040–0.045)		
Model 1^a^	0.928	0.919	0.044 (0.043–0.046)		
Model 2^b^	0.937	0.932	0.040 (0.039–0.042)	Model 2 vs 1	0.009
Model 3^c^	0.928	0.926	0.042 (0.041–0.044)	Model 3 vs 2	0.009
Preschool teachers					
Child gender^§^					
Girls (*n* = 2596)	0.944	0.936	0.045 (0.043–0.048)		
Boys (*n* = 2770)	0.957	0.951	0.052 (0.050–0.054)		
Model 1^a^	0.952	0.946	0.049 (0.047–0.050)		
Model 2^b^	0.960	0.957	0.044 (0.042–0.045)	Model 2 vs 1	0.008
Model 3^c^	0.957	0.955	0.044 (0.043–0.046)	Model 3 vs 2	0.003
Child age					
3-year-olds (*n* = 1828)	0.955	0.949	0.049 (0.046–0.052)		
4-year-olds (*n* = 1710)	0.950	0.944	0.051 (0.048–0.054)		
5-year-olds (*n* = 1829)	0.967	0.962	0.040 (0.037–0.043)		
Model 1^a^	0.957	0.951	0.047 (0.045–0.048)		
Model 2^b^	0.965	0.962	0.041 (0.040–0.043)	Model 2 vs 1	0.008
Model 3^c^	0.960	0.959	0.043 (0.041–0.045)	Model 3 vs 2	0.005

*CFI* Comparative Fit Index, *TLI* Tucker-Lewis Index, *RMSEA* root mean square error of approximation

CFI or TLI > 0.90 and RMSEA < 0.06 indicate acceptable fit; Change in CFI (∆ CFI) used as criteria test differences between two nested models—∆ CFI less than 0.01 indicates *p* > .05 (not significant)

^a^All parameters free

^b^Constrained factor loadings

^c^Constrained factor loadings and intercepts

^†^3 cases with missing gender information

^‡^8 cases with missing gender information

^§^1 case with missing gender information

**Table 5:** Model fit and nested model comparisons for multiple-group CFA: parental education level

Multiple group CFA	Model Fit Indices			Nested model comparisons	
	CFI	TLI	RMSEA	Comparison	Change in CFI
Fathers					
Lower education (*n* = 2630)	0.917	0.907	0.048 (0.046–0.050)		
Higher education (*n* = 2827)	0.914	0.903	0.048 (0.046–0.050)		
Model 1^a^	0.916	0.905	0.048 (0.046–0.049)		
Model 2^b^	0.923	0.916	0.045 (0.043–0.046)	Model 2 vs 1	0.008
Model 3^c^	0.919	0.915	0.045 (0.044–0.047)	Model 3 vs 2	0.004
Mothers					
Lower education (*n* = 2331)	0.924	0.914	0.048 (0.046–0.051)		
Higher education (*n* = 3991)	0.930	0.921	0.043 (0.042–0.045)		
Model 1^a^	0.926	0.917	0.045 (0.044–0.047)		
Model 2^b^	0.932	0.926	0.043 (0.041–0.044)	Model 2 vs 1	0.006
Model 3^c^	0.928	0.924	0.043 (0.042–0.045)	Model 3 vs 2	0.004

*CFI* Comparative Fit Index, *TLI* Tucker-Lewis Index, *RMSEA* root mean square error of approximation

CFI or TLI > 0.90 and RMSEA < 0.06 indicate acceptable fit; Change in CFI (∆ CFI) used as criteria test differences between two nested models—∆ CFI less than 0.01 indicates *p* > .05 (not significant)

^a^All parameters free

^b^Constrained factor loadings

^c^Constrained factor loadings and intercepts

To assess MI across 3-, 4-, and 5-year-olds, similar procedures were undertaken, with separate analyses for fathers, mothers and preschool teachers. No significant changes in CFI were found when testing for metric invariance (Table 4). Likewise, with additional restrictions on intercepts, scalar invariance was considered to be present. The results implied MI across child age groups for fathers, mothers, and preschool teachers.

Next, MI across education level groups of mothers and fathers was assessed, using the same procedures as above. Both metric and scalar invariance were established, based on non-significant changes in CFI. See Table 5 for fit measures and model comparisons.

## Discussion

The purpose of this study was to investigate the factor structure of the SDQ for fathers, mothers and preschool teachers in a community sample, as well as to assess measurement invariance across child gender, child age, and parental education level. Our study adds to the psychometric literature on the SDQ and specifically increases knowledge about the construct validity of the SDQ when rating pre-schoolers. Previous research has mainly focused on school-aged children and adolescents and rarely on preschool children only, making our study a new and valuable contribution to knowledge on the SDQ for this age group by thoroughly assessing the factor structure across different informants and confirming MI. Our findings show that the original five-factor model of the SDQ can indeed be used on younger children in a general child population, also indicating its reliability in the hands of different informants.

This is the first time that data from fathers have been analysed separately through CFA when assessing the construct validity of the SDQ for pre-schoolers, and the second time for all ages [19]. Fathers are increasingly involved in and important for the child’s development and can provide very useful information about the child [38]. Still, psychometric properties of questionnaires like the SDQ that measure children’s behaviour and emotional problems have seldom been investigated for fathers. To secure gender equality at child health services concerning involvement of both parents, it is essential to use instruments that are validated for fathers as well as mothers.

In addition, preschool teachers as informants have not been thoroughly assessed before, making this study an important contribution to aggregated knowledge on the SDQ. It is also an important step towards gathering reliable information from multiple sources when assessing children’s mental health, as recommended by Goodman et al. [4, 39]. In a study by Fält et al. [40], the child health nurses reported that the quality of health check-ups for 3–5-year-olds improved when they had SDQ ratings from the preschool teacher as well as from the parents. The present study adds to the knowledge on the SDQ as a valuable tool for screening for mental illness in children by assessing its construct validity when used on preschool children. Since 95% of all preschool children in Sweden visit child health centres regularly [26] and attend preschool, using the SDQ as the standard procedure in this setting could be one way of reaching children at risk at an early stage.

The invariance analyses suggest that both metric and scalar invariances are present for all informants, across child gender, child age, and education levels of the parents. MI has only been established across different countries [41] and informant groups [22]. Although Stone et al. [23] provide some evidence for metric invariance across child gender and maternal education level for mothers rating school children, the cut-offs that they used for acceptable fit were more liberal than generally recommended. Our study shows metric invariance according to more conservative standards.

The results from our study confirm the five-factor structure for the Swedish version of the SDQ, thus being in line with previous studies suggesting that the SDQ is comparable across cultures [21, 42]. The results also indicate that cultural modification of the SDQ, in terms of an altered wording of three items, based on preschool organisational preferences, did not jeopardise the model fit.

Although the factor structure was acceptable for mothers, fathers and preschool teachers alike, we were not able to test for MI across informants. This was due to the fact that many children in the current sample were rated by more than one informant. Thus, clustering effects were likely to occur in a random fashion, for which we were not able to control. Therefore, we decided to analyse the data from the three informants separately, resulting in as much data as possible and not introducing possible confounders by removing children not rated by all informants. Comparison of fitness between informants was therefore not possible, which can be seen as a limitation.

The sample in our study included a larger proportion of people with higher education compared to the distribution within the municipality. Although we cannot completely eliminate the possibility that the slightly lopsided sample in terms of parental education level could affect the factor structure, MI across the education variable suggests that the SDQ is a valid instrument across different parental education levels.

As the SDQ shows satisfactory construct validity, it would be desirable to present norms for this age group and for all three informants in a future study. In fact, to facilitate the application of the SDQ in clinical settings, it is necessary to provide norms from a large community sample, covering children aged 3–5 and all three informants. Finally, although we found the construct validity to be good in the present study, we did not analyse any competing theoretical models of the factor structure of the SDQ. It is possible that another theoretical model would provide even better fit, such as the four-factor multi-trait-multi-method structure suggested by Bull et al. [43]. However, since the original model is the one being used in clinical practice and the only model provided with norms, we chose to analyse this model only.

Notwithstanding the above-mentioned limitations, the results from our study imply that the SDQ is a feasible instrument for assessing emotional and behavioural problems among preschool children. Moreover, our study is an important step in investigating whether the original five-factor structure of the SDQ can be used in large community samples. Findings based on fathers’ ratings suggest acceptable fit, implying that the SDQ rated by fathers can be used to the same extent as mothers’ ratings to assess behaviour problems among preschool children.

Lastly, the sample in this study was a non-clinical sample, which allowed for assessment of the construct validity of the instrument when used on the general population. Factor analytic studies from the lengthier but comparable CBCL for preschool children indicate good fit when rated by parents or preschool teachers in clinical and non-clinical samples in most but not all studies [44–47]. However, CFAs assessing fathers’ ratings have not examined the questionnaire on subscale-level [48]. Our findings suggest that the SDQ might be a suitable instrument for epidemiological studies of preschool children’s mental health and can be used in its originally proposed five-factor solution in non-clinical populations. This enables thorough investigation of subscale differences in epidemiological studies, thus resulting in more detailed studies of populations.

## Summary

The SDQ is a well-established instrument for measuring social and behavioural problems among children, with good psychometric properties for older children, but less validity reports on pre-schoolers. In addition, there is a knowledge gap concerning fathers as informants. The present work is one of the few validity studies to include preschool teachers and the first on preschool children, where fathers are included as separate informants. In this study, the SDQ was collected from a large community sample (*n* = 17,752) of children aged 3–5, rated by mothers, fathers, and preschool teachers and analysed using alpha calculations and confirmatory factor analysis. Measurement invariance analyses were also conducted to assess invariance across child gender, child age, and parental education level. Our results revealed high internal consistency, acceptable fit for all informant groups and measurement invariance across child gender, child age, and parental education level. Our findings suggest good construct validity of the SDQ for a non-clinical preschool population and imply that it may be used for assessing behaviour problems in pre-schoolers from different informant perspectives.
